# Identifying enhancer-driven subtype-specific prognostic markers in breast cancer based on multi-omics data

**DOI:** 10.3389/fimmu.2022.990143

**Published:** 2022-10-11

**Authors:** Hongying Zhao, Siwen Zhang, Xiangzhe Yin, Caiyu Zhang, Lixia Wang, Kailai Liu, Haotian Xu, Wangyang Liu, Lin Bo, Shihua Lin, Ke Feng, Lin Lin, Meiting Fei, Shangwei Ning, Li Wang

**Affiliations:** College of Bioinformatics Science and Technology, Harbin Medical University, Harbin, China

**Keywords:** breast cancer subtype, enhancer, lncRNA, copy number variation, prognostic marker

## Abstract

Breast cancer is a cancer of high complexity and heterogeneity, with differences in prognosis and survival among patients of different subtypes. Copy number variations (CNVs) within enhancers are crucial drivers of tumorigenesis by influencing expression of their targets. In this study, we performed an integrative approach to identify CNA-driven enhancers and their effect on expression of target genes in four breast cancer subtypes by integrating expression data, copy number data and H3K27ac data. We identified 672, 555, 531, 361 CNA-driven enhancer-gene pairs and 280, 189, 113 and 98 CNA-driven enhancer-lncRNA pairs in the Basal-like, Her2, LumA and LumB subtypes, respectively. We then reconstructed a CNV-driven enhancer-lncRNA-mRNA regulatory network in each subtype. Functional analysis showed CNA-driven enhancers play an important role in the progression of breast cancer subtypes by influencing P53 signaling pathway, PPAR signaling pathway, systemic lupus erythematosus and MAPK signaling pathway in the Basal-like, Her2, LumA and LumB subtypes, respectively. We characterized the potentially prognostic value of target genes of CNV-driven enhancer and lncRNA-mRNA pairs in the subtype-specific network. We identified MUM1 and AC016876.1 as prognostic biomarkers in LumA and Basal-like subtypes, respectively. Higher expression of MUM1 with an amplified enhancer exhibited poorer prognosis in LumA patients. Lower expression of AC016876.1 with a deleted enhancer exhibited poorer survival outcomes of Basal-like patients. We also identified enhancer-related lncRNA-mRNA pairs as prognostic biomarkers, including AC012313.2-MUM1 in the LumA, AC026471.4-PLK5 in the LumB, AC027307.2-OAZ1 in the Basal-like and AC022431.1-HCN2 in the Her2 subtypes. Finally, our results highlighted target genes of CNA-driven enhancers and enhancer-related lncRNA-mRNA pairs could act as prognostic markers and potential therapeutic targets in breast cancer subtypes.

## Introduction

Breast cancer is the most common cancer in women, with more than 1,300,000 cases and 450,000 deaths worldwide each year (1). Previous studies have shown that breast cancer is a cancer of high complexity and heterogeneity. The currently accepted classical classification of breast cancer uses microarray-based breast cancer tumor gene expression profiles to classify breast cancer into four intrinsic subtypes: Basal-like, Her2, Luminal A (LumA), and Luminal B (LumB) (1, 2). Different breast cancer subtypes have different molecular characteristics, clinical responses, and the effects of prognostic survival effects (3). Therefore, identifying the specific key regulators in different breast cancer subtypes can provide new ideas for understanding the occurrence and development mechanisms and clinical treatment of different subtypes.

Enhancers, a class of key noncoding regulatory DNA elements, have received increasing attention for their role in cancer development (4). Enhancers are scattered in 98% of non-coding protein genes in the human genome (5). Inactive enhancers in the human genome are tightly bound by unmodified nucleosomes, when the enhancer is activated, local chromatin is modified (usually by H3K4me1) and TF binds to the enhancer. Then when the enhancer is fully activated, the enhancer is re-marked by H3K27ac and recruits the RNA polymerase to initiate bidirectional transcription of genes (6). Thus, in the human genome, active enhancers have the histone modification signature of H3K27ac (7, 8). High-throughput epigenomics suggests that there are more than one million putative enhancers in the human genome, significantly exceeding protein-coding genes (9). Although transcription is a universal property of all cells, cancer cells are more dependent on increased transcription levels from enhancers (10). Copy number variations (CNVs) are probably most frequent genomic alteration events (11). Previous studies showed a close correlation between gen copy number variation and differential gene expression across many cancer types. Recent studies showed that alterations within enhancers are crucial drivers of tumorigenesis by influencing expression of their targets (12–14). For example, copy number amplification of enhancer elements caused the upregulation of oncogene MYC expression, which promotes malignant transformation of pediatric neuroblastomas (15). These studies suggest that aberrant alterations in enhancer can influence cancer progression by affecting target genes (16).

Studies suggest that disease-associated variants were significantly enriched in enhancer regulatory elements (17, 18) and CNVs could affect the enhancer regulation and altering the transcriptional regulation of downstream key target genes (13, 19). In a previous study, the investigators found that HOXC6 and DLX1 associate with different prostate tumor-specific enhancer clusters and confer distinct transcriptomic changes on C42B prostate cancer cells, which play a role in prostate tumor development (20). They also found that GATA3, which is linked to the enhancer, was overexpressed in non-basal-like breast tumors and affected the occurrence and development of breast cancer (20). Although lncRNAs do not encode proteins, many studies have shown that a small fraction of lncRNAs still function independently of the DNA sequences that transcribe them (21). Overexpression of LINC02095 promotes the proliferation of triple-negative breast cancer cells by promoting the expression of the oncogenic transcription factor SOX9, which promotes tumor activity (22). LINC00908 inhibits triple-negative breast cancer by regulating angiogenesis and tumor growth (23). The strong spatial and temporal specificity of lncRNA expression suggests that there may be a potential interaction between enhancers and lncRNAs (24). The binding of transcription factors NKX3.1 and YY1 to the PCAT19 promoter leads to strong enhancer activity and activation of lncRNA PCAT19, thereby promoting tumor growth and metastasis of prostate cancer (25). Therefore, an in-depth understanding of the regulatory relationship and functional relationship between enhancers, lncRNAs and mRNAs is of great significance for us to explore the carcinogenic mechanism of breast cancer, as well as to find novel and effective prognostic markers as therapeutic targets.

This study characterized the effect of copy number variation-driven enhancers and their affected biological functions in breast cancer occurrence and progression by integrating TCGA breast cancer multi-omics data. We identified CNA-driven enhancers and their affected differentially expressed genes and lncRNAs in the Basal-like, Her2, LumA and LumB breast cancer subtypes. We then reconstructed the subtype-specific enhancer-lncRNA-mRNA regulatory networks and performed their prognostic analysis. Finally, in the LumA subtype, MUM1 and the AC012313.2-MUM1 pair were identified as prognostic biomarkers. In the LumB subtype, the AC026471.4-PLK5 pair was identified as a prognostic biomarker. In the Basal-liked subtype, AC016876.1 and the AC027307.2-OAZ1 pair were identified as prognostic biomarkers. In the Her2 subtype, the AC022431.1-HCN2 pair was identified as a prognostic biomarker. Our results highlighted the important role of target genes of CNA-driven enhancers and enhancer-related lncRNA-mRNA pairs in clinical diagnosis and treatment of breast cancer patients.

## Results

### Identification of copy number variation-driven enhancers in breast cancer

**Figure 1:**
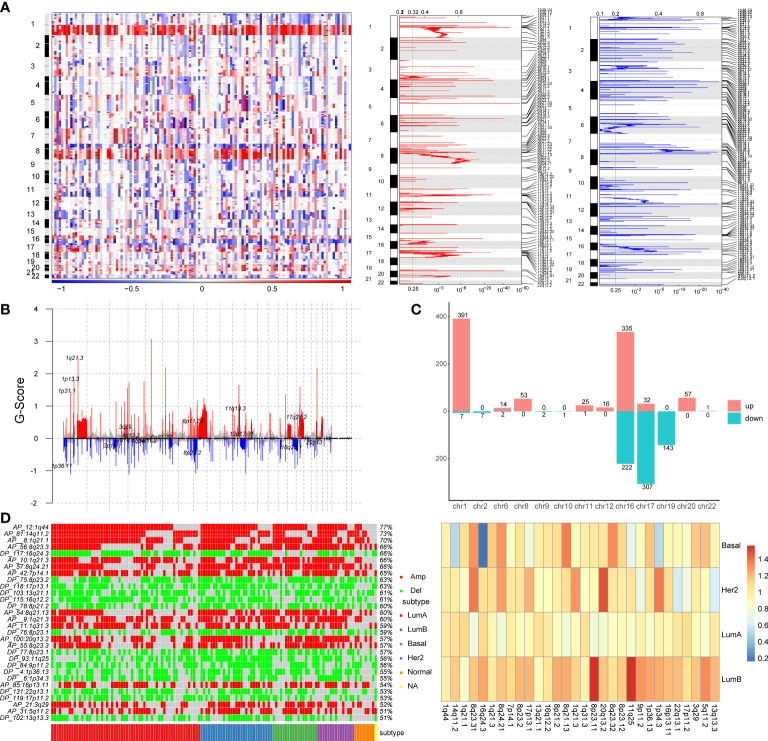
Identification of copy number variation-driven enhancers in breast cancer. **(A)** The analysis of copy number variation in breast cancer by GISTIC2.0. The left panel shows CNV variation heatmap, the middle panel shows copy number amplification regions and the right panel shows copy number deletion regions. **(B)** Waterfall plot of CNV rates in breast cancer. The red line indicates copy number amplification and the blue line indicates copy number deletion. **(C)** Chromosomal distribution of copy number variation-driven enhancers. **(D)** Comparative analysis of significant copy number variation regions in four subtypes of breast cancer. The left panel shows illustrates the variation of the significant CNV region in four subtypes and the overall mutation rate of these CNV region. The right panel shows a heatmap of folds of CNV mutation rate of each subtypes and total mutation rate.

To explore the effect of copy number variation on enhancer elements in breast cancer, we firstly identify genomic regions that are significantly amplified or deleted across breast cancer samples using GISTIC2.0 algorithm. We found that a large number of copy number alterations were detected, with amplification of part of chromosomes 1q, 8q, 16p, and 17q, and deletion of part of chromosomes 8p, 16q, and 17p (**Figure 1A**). Copy number variation chromosome maps also validated the copy number variation profile of these regions (26, 27)(**Figure 1B**). At the same time, we found that some chromosomes showed the same variation trend on the entire chromosome, such as the overall copy number amplification of chromosome 3, the overall copy number loss of chromosome 4, but other chromosomes such as chromosomes 1, 8 and 16 showed the opposite trend of copy number variation at arm-level (**Figure 1B**). For example, the chromosome 1q exhibited significant copy number expansion, while the chromosome 1p did not.

Subsequently, in order to identify enhancers in breast cancer, we obtained H3K27ac histone modification data in breast cancer cell lines MCF-7 from the Cistrome database. We obtained 20,805 active enhancer sites using histone modification marker H3K27ac (MACS2, q-value=0.01). Then we mapped significant copy number variation to active enhancer sites using BEDtools to identify copy number variation-driven (CNV-driven) enhancers, we finally obtained 1617 CNV-driven enhancers in breast cancer, of which 925 active enhancers were driven by CNA amplification and 692 active enhancers were driven by CNA deletion (**Figure 1C**). A large number of amplified enhancers were located on chromosomes 1 and 16. Most of deleted enhancers were located on chromosomes 16, 17 and 19. In addition, to explore the differences of copy number variation among breast cancer subtypes, we compared the differences of copy number variation in significant CNV regions in breast cancer subtypes, and the differences of copy number variation between each subtype and all subtypes (**Figure 1D**). The heatmap showed that there are obvious copy number variations in breast cancer, for example, the mutation rate in the 1q44, 14q11.2 and 1q21.1 regions exceeds 70% (**Figure 1D**). At the same time, we found that some regions, such as the 1q44, 14q11.2 and 1q21.1 regions, showed obvious amplification in most samples of four subtypes, while some regions, such as 7p14.1, 11q25 and 20q13.2 regions, showed significantly higher mutation rate in LumB and the Her2 subtypes than in other subtypes. We also found that the copy number variation rate of the 16q24.3 region was significantly lower in the Basal-like subtype than in other subtypes (**Figure 1D**). By comparing the variation rate of each subtype with the overall variation rate, we found that the variation rate of the LumB subtype was generally higher than the overall mutation rate, while the variation rate of the LumA subtype was lower than the overall mutation rate (**Figure 1D**). These findings provided evidence that copy number variants (CNVs) were associated with breast cancer subtypes (28).

### Constructing subtype-specific regulations between CNV-driven enhancers and lncRNAs and mRNAs

**Figure 2:**
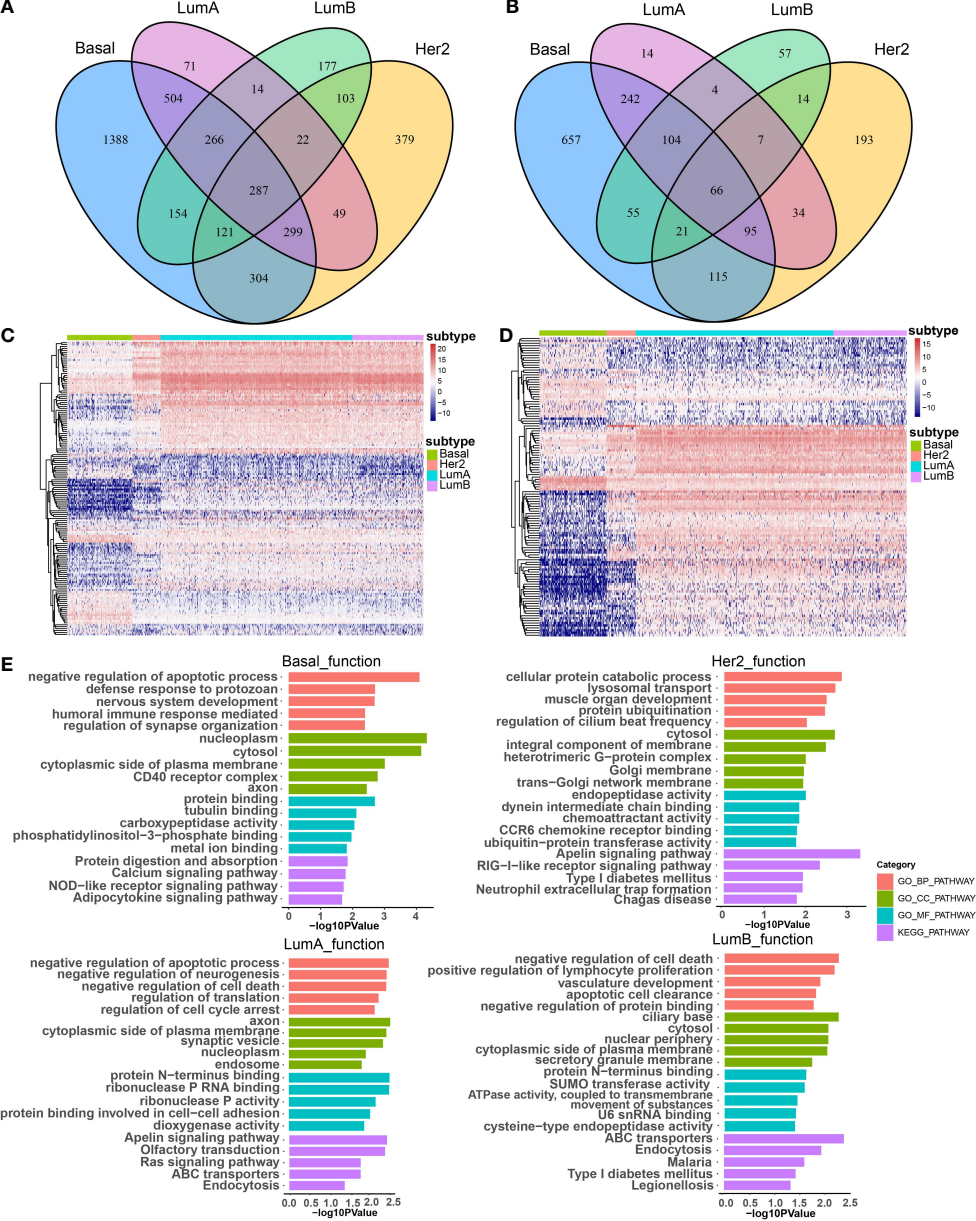
The effect of CNA-driven enhancers on expression of target genes. Venn diagram of differential genes **(A)** and differential lncRNAs **(B)** in the four subtypes. **(C)** Differential gene expression profiles of the four subtypes, and the differential gene set is the sum of the four subtype-specific differential gene sets. **(D)** Differential lncRNA expression profiles of the four subtypes, and the differential lncRNA set is the sum of the four subtype-specific differential lncRNA sets. **(E)** The top-ranked terms of GO function and KEGG pathway for differentially expressed genes in Basal-like, her2, LumA and LumB subtypes using DAVID.

To investigate the effect of CNA-driven enhancers on expression of target genes in the four breast cancer subtypes, we firstly identified subtype-specific differentially expressed genes (DEGs) and differentially expressed lncRNAs (DELs), with a threshold of FDR < 0.05 and logFC > 1 in the Basal-like, Her2, LumA, LumB subtypes, respectively (**Figures 2A, B**). Comparative analysis between subtypes showed that the Basal-like subtype showed a distinct and unique expression pattern of genes and lncRNAs, with an overall trend towards low expression, the LumA and LumB subtypes had very similar expression patterns, with an overall trend towards high expression, and the expression pattern of genes and lncRNAs in Her2 subtype was not biased (**Figures 2C, D**). Furthermore, we performed GO function and KEGG pathway enrichment analysis on the differentially expressed genes of the four breast cancer subtypes using the DAVID database (**Figure 2E**). The results show that the four subtypes affect some specific biological functions and pathways. For example, in the Basal-like subtype, differentially expressed genes were enriched in the humoral immune responses, calcium signaling pathways and NOD-like receptor signaling pathway. In the Her2 subtype, differentially expressed genes were enriched in chemotactic activity and CCR6 chemokine receptor. In the LumA subtype, differentially expressed genes were enriched in the regulation of cell cycle arrest and the Ras signaling pathway. In the LumB subtype, differentially expressed genes were enriched in ATPase activity and the development of the vasculature. In addition, common differentially expressed genes shared by the four subtypes were enriched in protein binding or protein catabolism. Basal-like, LumA and LumB subtypes were enriched in the regulation of apoptosis, the regulation of cell death and the clearance of apoptotic cells. The main biological functions and pathways affected by LumA and LumB subtypes were more similar than other subtypes, for example, both were enriched in negative regulation of cell death, apoptotic process, protein N-terminus binding, ABC transporters and Endocytosis. These functions are critical in the survival, invasion, proliferation and immune escape of breast cancer cells. Different subtypes shared some biological functions, but also have many specific biological functions, which lead to differences in cancer development and outcomes among subtypes.

**Figure 3:**
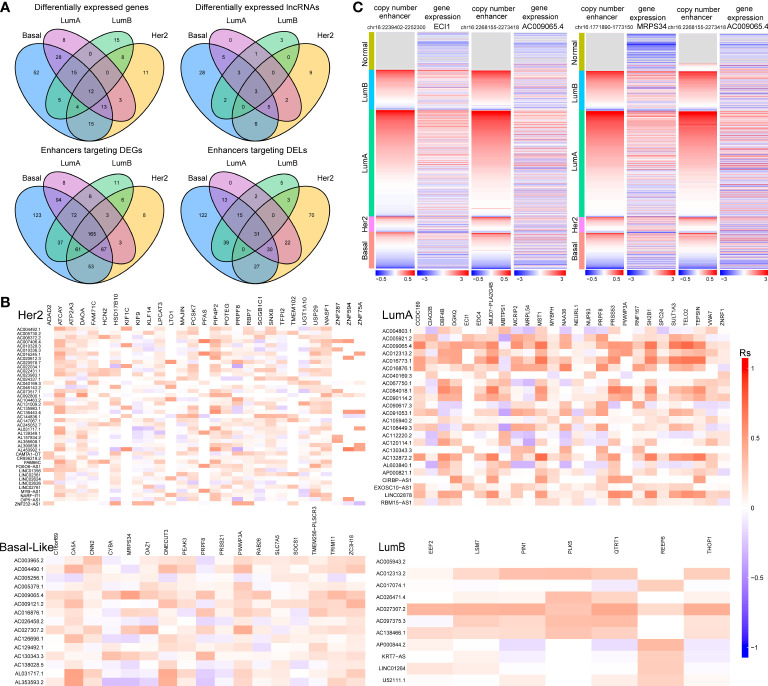
The relationship between CNV-driven enhancer target genes and related lncRNAs **(A)** Venn diagram analysis of CNV-driven enhancer target genes and related lncRNAs of four subtypes of breast cancer. The top panel shows the CNV-driven enhancer target genes and related lncRNAs, respectively, in the four subtypes. The bottom panel shows the enhancers of DEGs and the enhancers of DELs in the four subtypes. **(B)** Correlation analysis of differentially expressed genes and differentially expressed lncRNAs in four subtypes of breast cancer. X-axis is symbol of DEGs and y-axis is symbol of DELs. **(C)** Copy number variation of enhancers (chr16:2239402-2252300; chr16:1771890-1773150; chr16:2268155-2273418) and expression levels of ECI1, MRPS34 and AC009065.4 in breast cancer samples.

Additionally, we found that there was a significant correlation between tumour subtypes and pathological stage of breast cancer (**Figure S1A**). For example, the proportion of patients with stage I breast cancer was significantly higher in the LumA subtype (P=0.01, chi-square test). A significantly higher proportion of patients with stage IV breast cancer were observed in the Her2 subtype (P=0.03, chi-square test, **Figure S1A**). For each subtype, subtype-specific DEGs (or DELs) within 100 kb of CNV-driven enhancers and showing consistency in CNAs with gene expression were identified as subtype-specific enhancer-gene (or enhancer-lncRNA) pairs (29, 30). As a result, we identified 672 enhancer-gene pairs and 280 enhancer-lncRNA pairs in the Basal-like subtype, 555 enhancer-gene pairs and 189 enhancer-lncRNA pairs in the Her2 subtype, 531 enhancer-gene pairs and 113 enhancer-lncRNA pair in the LumA subtype, and 361 enhancer-gene pairs and 98 enhancer-lncRNA pairs in the LumB subtype (**Table S1**). Comparative analysis revealed that the four cancer subtypes shared many common target genes and lncRNAs of CNA-driven enhancers (**Figure 3A**). At the same time, we noticed that in the enhancer-lncRNA regulatory relationship pair, the LumA subtype did not have specific enhancers and lncRNAs, but shared many enhancers and lncRNAs with the Basal-like subtype (**Figure 3A**). For example, AC009065.4 which is driven by copy number-amplified enhancers is found to be differentially expressed in LumA and Basal-like subtypes. Furthermore, we identified co-expressed lncRNA-mRNA pairs (P<0.05) to characterize the correlation between DELs and DEGs in each breast cancer subtype. We found that most of the lncRNA-mRNA pairs showed subtype-specific regulations, even for genes shared by several subtypes (**Figure 3B**). For example, in the LumA subtype, AC009065.4 is co-expressed with a key mitochondrial fatty acid β-oxidation enzyme ECI1 (R=0.45, P<0.001). AC009065.4 was upregulated in the LumA subtype (logFC=1.67, P=6.12e-30) which is mediated by a CNV-amplified enhancer (chr16:2268155-2273418). Its co-expressed gene ECI1 was also upregulated in the LumA subtype (logFC=1.13, P=3.79e-10) which is mediated by a CNV-amplified enhancer (chr16:2239402-2252300; **Figure 3C**). Studies showed that over-expression of ECI1 are related with the risk of distant metastasis and reduced survival in prostate cancer patients (31). In the Basal-like subtype, AC009065.4 and MRPS34 were significantly co-expressed (R=0.48, P=4.82e-13). AC009065.4 was downregulated in the Basal-like subtype (logFC=-1.40, P=3.28e-36) which is mediated by a CNV-deleted enhancer (chr16:2268155-2273418). Its co-expressed gene MRPS34 was also downregulated in the Basal-like subtype (logFC=1.13, P=3.79e-10) which is mediated by a CNV-deleted enhancer (chr16:1771890-1773150; **Figure 3C**). It is reported that dysregulated gene expression of MRPS34 was related with cancer cell stemness and chemoresistance in cholangiocarcinoma (32).

### Reconstruction and functional characterization of subtype-specific CNV-driven enhancer-lncRNA-mRNA network

**Figure 4:**
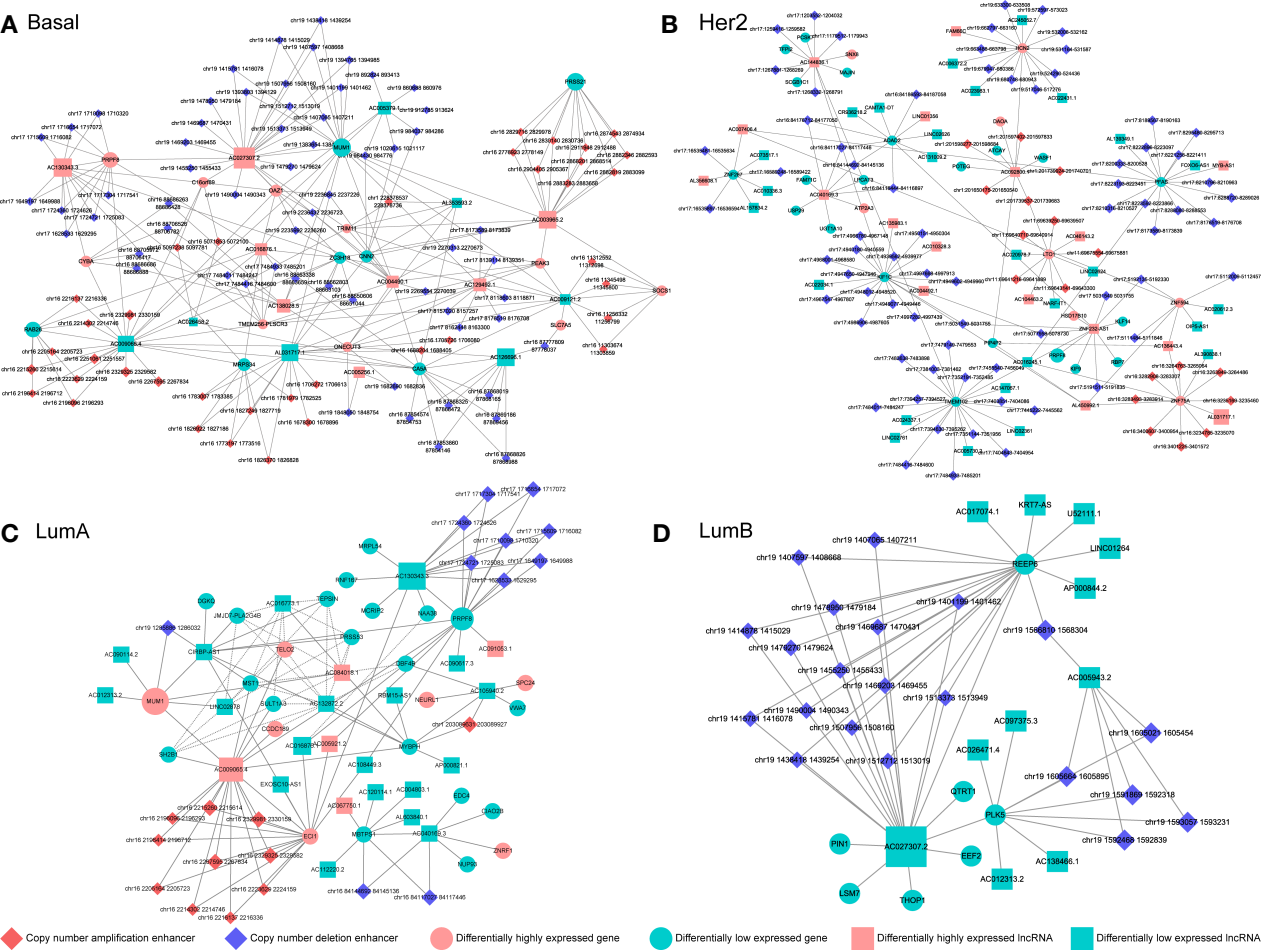
The CNV-driven enhancer-lncRNA-mRNA networks in Basal-like subtype **(A)**, Her2 subtype **(B)**, LumA subtype **(C)** and LumB subtype **(D)**. The diamond represents CNA-driven enhancers (red: amplification; blue: deletion). The circle and square indicate differentially expressed genes and lncRNAs, respectively. Pink denotes upregulation and green denotes downregulation.

**Figure 5:**
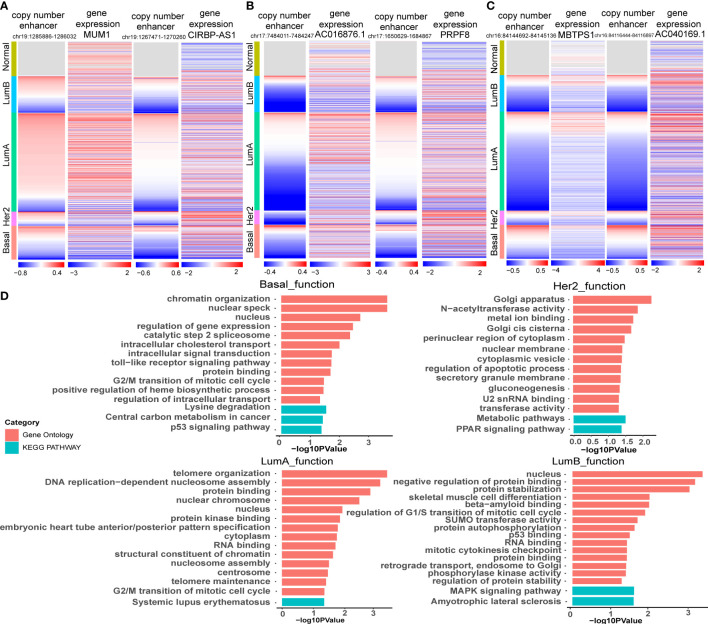
Functional enrichment analysis of target genes of CNA-driven enhancers in breast cancer subtypes. **(A)** Copy number variation of enhancers (chr19:1285886-1286032; chr19:1267471-1270260) and expression levels of MUM1 and CIRBP-AS1 in breast cancer samples. **(B)** Copy number variation of enhancers (chr17:7484011-7484247; chr17:1650629-1684867) and expression levels of AC016876.1 and PRPF8 in breast cancer samples. **(C)** Copy number variation of enhancers (chr16:84144692-84145136; chr16:84116444-84116897) and expression levels of MBTPS1 and AC040169.1 in breast cancer samples. **(D)** GO function and KEGG pathway analysis for key genes in the CNA-driven enhancer-lncRNA-mRNA network in Basal-like, her2, LumA and LumB subtypes of breast cancer using DAVID. The significance threshold was FDR less than 0.05.

We reconstructed a subtype-specific enhancer-lncRNA-mRNA regulatory network (ELMN) in each subtype by integrating CNA-driven enhancers, enhancer-genes, enhancer-lncRNAs and lncRNA-mRNA pairs. As a result, there were 100 CNV-driven enhancers, 17 DEGs and 15 DELs in the ELMN of Basal-like subtype (**Figure 4A**), 88 CNV-driven enhancers, 29 DEGs and 43 DELs in the ELMN of Her2 subtype (**Figure 4B**), 22 CNV-driven enhancers, 26 DEGs and 24 DELs in the LumA subtype (**Figure 4C**) and 21 CNV-driven enhancers, 7 DEGs and 11 DELs in the LumB subtype (**Figure 4D**). For example, PWWP domain-containing protein MUM1 (also known as EXPAND1) is significantly co-expressed with lncRNA CIRBP-AS1 in LumA subtype of breast cancer. MUM1 was upregulated in the LumA subtype which is mediated by a CNV-amplified enhancer (chr19:1285886-1286032). Its co-expressed lncRNA CIRBP-AS1 was also upregulated in the LumA subtype which is mediated by a CNV-amplified enhancer (chr19:1267471-1270260; **Figure 5A**). Elevated expression of CIRBP-AS1 was reported to be related with poor prognosis in breast cancer and lower-grade gliomas (33). In the Basal-like subtype, lncRNA AC016876.1 was identified to be significantly co-expressed with PRPF8. AC016876.1 was downregulated in the Basal-like subtype which is mediated by a CNV-deleted enhancer (chr17:7484011-7484247). PRPF8 was also downregulated in the Basal-like subtype which is mediated by a CNV-deleted enhancer (chr17:1650629-1684867; **Figure 5B**). It was reported that PRPF8 knockdown could lead to widespread intronic retention and altered splicing of transcripts including protein homeostasis, mitosis, and apoptosis in the Basal-like TNBC (34).

The enhancer-gene-lncRNA triplet is considered to be involved in the occurrence and development of breast cancer as a relatively stable functional unit in subtypes. In the LumA subtype, there are two enhancer-lncRNA-mRNA triples, including enhancer(chr16:84144692-84145136)-AC040169.1-MBTPS1 and enhancer(chr16:84117027-84117446)-AC040169.1-MBTPS1, were found in the ELMN (**Figure 4C**). Enhancers (chr16:84117027-84117446 and chr16:84144692-84145136) showed copy number loss in LumA subtype of breast cancer. Their target genes MBTPS1 and AC040169.1 were significantly decreased in the LumA subtype as compared to the other subtypes (**Figure 5C**). MBTPS1 was significantly co-expressed with AC040169.1 in the LumA subtype (R= 0.24; P= 4.75e-9). LncRNA AC040169.3 transcribed from the intronic region of MBTPS1. Previous studies showed that the decreased expression of MBTPS1 produced by chromosome 16q loss were reported in the oestrogen receptor (ER)-positive breast cancer (35). It may suggest that enhancer-AC040169.1-MBTPS1 triples could be associated with the risk of LumA breast cancer.

Functional enrichment analysis of the genes in the specific-subtype CNV-driven ELMN showed that the top significant GO terms in the Basal-like subtype regulatory network were regulation of gene expression, protein binding, cell cycle regulation and the top KEGG pathways were P53 signaling pathway and central carbon metabolism in cancer (**Figure 5D**). In the Her2 subtype, the top significant GO term was regulation of apoptotic process, and the top KEGG pathway was the PPAR signaling pathway. In the LumA subtype, the top GO terms were regulation of cell cycle regulation, protein binding, DNA replication-dependent nucleosome assembly and the top KEGG pathway was systemic lupus erythematosus. In the LumB subtype, the top significant GO terms were regulation of G1/S transition of the mitotic cell cycle, P53 binding, regulation of protein stability, and the top KEGG pathway was MAPK signaling pathway. The four subtypes were mainly related to the cell cycle, apoptosis, protein binding and other functions, which may affect the cell proliferation and the occurrence and development of breast cancer (36).

### Subtype-specific prognostic biomarkers mediated by CNV-driven enhancer in breast cancer

**Figure 6:**
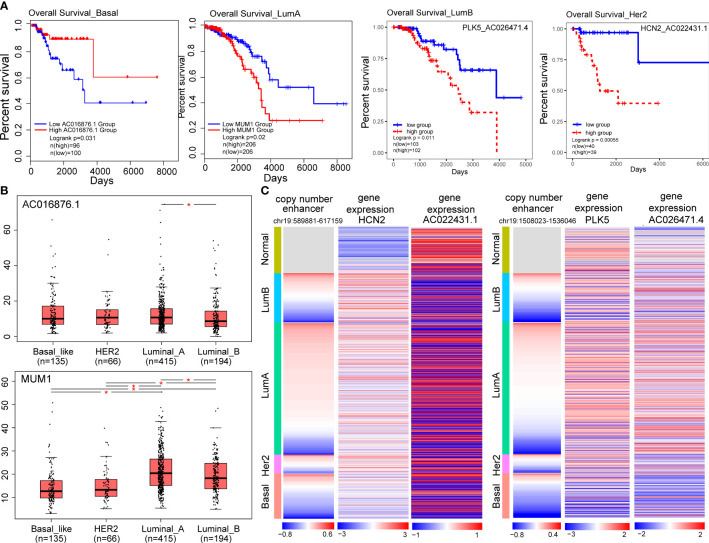
Target genes of CNA-driven enhancers elements and lncRNA-mRNA pairs are associated with prognosis in breast cancer subtypes. **(A)** Kaplan-Meier survival curves according to the gene expression or risk score of lncRNA-mRNA in breast cancer subtypes. The log-rank test was used to estimate the difference in survival time. **(B)** The differential expression of prognostic markers across the four subtypes using one-way ANOVA. (*p < 0.05). **(C)** Copy number variation of enhancers (chr19:589881-617159; chr19:1508023-1536046) and expression levels of HCN2, PLK5 and their co-expressed genes in breast cancer samples.

To explore the potentially prognostic value of target genes of CNV-driven enhancer and lncRNA-mRNA pairs in the subtype-specific ELMN, we performed survival analysis. We found MUM1 in the LumA subtype, lncRNA AC016876.1 in the Basal-like subtype were able to significantly distinguish patients in high-risk groups from those in low-risk groups in terms of overall survival (**Figure 6A**; **Table 1**). For example, MUM1 with an amplified enhancer was significantly highly expressed in LumA subtype when comparing with the other subtypes (P=2.7e-4, chi-square test; **Figure S1B**). Survival analysis showed that higher expression of MUM1 was associated with poorer prognosis in LumA subtype (P<0.05; **Figure 6A**). It is reported that MUM1 was involved in DNA repair and the regulation of DNA damage. Previous studies showed that an interaction partner of EXPAND1, the p53-binding protein-1 (53BP1) showed higher expression in luminal A breast cancer cell line MCF-7 and T47D when comparing to MDA-MB-468 and MDA-MB-231 cell lines (37, 38). Knockdown of 53BP1 is reported to significantly enhance the growth of luminal A breast cancer MCF-7 cells, which may suggest that the EXPAND1 could play important role in luminal A breast cancer by interacting with 53BP1. Thus, the expression of MUM1 increased is used as a poor prognostic marker in LumA patients. In the Basal-like subtype, AC016876.1 with a deleted enhancer was significantly down-expressed as compared to the other subtypes (logFC=-3.53, FDR =2.89e-82, DEseq2; **Figure 6B**; Wilcoxon test, P=2.2e-06, **Figure S1C**). Kaplan–Meier survival curve showed that patients with low expression of AC016876.1 were significantly associated with poor overall survival in Basal-like subtype (log-rank test, P= 0.03; **Figure 6A**). A previous study showed that AC016876.1 is associated with autophagy and can be used as a biomarker to effectively guide and predict the clinical prognostic effect of colorectal cancer patients (39). Thus, the expression of AC016876.1 decreased is used as a poor prognostic marker in Basal-like patients.

**Table 1:** Targets of CNA-driven enhancers and lncRNA-mRNA pairs as subtype-specific prognostic markers.

Subtype	Prognostic markers	CNA-driven enhancer	P value (log rank test)
LumA	MUM1	Copy number amplification	0.02
LumA	AC012313.2-MUM1	Copy number amplification	6.2e-4
LumB	AC026471.4-PLK5	Copy number deletion	0.01
Basal-like	AC016876.1	Copy number deletion	0.03
Basal-like	AC027307.2-OAZ1	Copy number deletion	0.01
Her2	AC022431.1-HCN2	Copy number amplification	5.5e-4

Additionally, we also identified enhancer-related co-expressed lncRNA-mRNA pairs as prognostic biomarkers, including one (AC012313.2-MUM1) in the LumA, one (AC026471.4-PLK5) in the LumB, one (AC027307.2-OAZ1) in the Basal-like and one (AC022431.1-HCN2) in the Her2 subtype (**Table 1**; **Figure 6A**). For example, a high-risk score of the PLK5-AC026471.4 signature in the LumB subtype had shortened survival (P=0.01, log-rank test; **Figure 6A**). LncRNA AC026471.4 was identified to be significantly co-expressed with PLK5 in the LumB subtype. Tumor-suppressor PLK5 was downregulated in the LumB subtype (log_2_ FC=-3.29; P=2.58e-69) which is mediated by a CNV-deleted enhancer (chr19:1508023-1536046). Its co-expressed lncRNA AC026471.4 was also downregulated in the LumB subtype (**Figure 6C**). It was reported that loss of heterozygosity and downregulation of PLK5 gene were frequently detected in cancer and its overexpression had an antiproliferative effect in cancer cell lines (40). In the Her2 subtype, a high-risk score of the HCN2-AC022431.1 signature was significantly associated with a worse prognosis (P= 5.5e-4, log-rank test; **Figure 6A**). LncRNA AC022431.1 was identified to be significantly co-expressed with HCN2 in the LumB subtype (R=0.30, P=5.8e-3). HCN2 was upregulated in the Her2 subtype (log_2_ FC=2.07, P=7.83e-15) which is mediated by a CNV-amplified enhancer (chr19:589881-617159). Studies have found that HCN2 showed the most significant upregulation in the HER2-positive and triple-negative breast cancer (TNBC) cell lines as compared to ER-positive breast cancer cell lines (41). Knockdown of HCN2 using shRNAs could significantly suppress proliferation of TNBC cell lines (41). Collectively, our findings underline the crucial roles of target genes of CNA-driven enhancer and enhancer-related lncRNA-mRNA pairs in breast cancer subtype carcinogenesis and their potential prognostic value.

## Methods

### Data

We obtained RNA-Seq expression profiles and copy number profiles of 1109 breast cancer patients from The Cancer Genome Atlas (TCGA) database (https://portal.gdc.cancer.gov/). Then we downloaded the whole gene annotation file of the human genome from the GENCODE database portal (https://www.gencodegenes.org). H3K27ac histone modification data in breast cancer cell line MCF-7 was obtained from the Cistrome database (42).

### Identification of copy number variation-driven active enhancers

We used the GISTIC2.0 algorithm (43) to identify genomic regions with significant copy-number variation (CNV) using SNP6.0 array-based copy number data for breast cancer from the TCGA database, H3K27ac ChIP-seq data was mapped to the reference genome GRCh38 using BWA (version 0.7.15) with default settings (44). Peak calling for H3K27ac ChIP-seq was performed using MACS2 with a q-value threshold of 0.01 (42). Significant H3K27ac peaks were defined as potential enhancers in breast cancer. Based on the genomic coordinates, CNV regions were mapped to enhancers using BEDTools. Enhancers with significant copy number variation (amplified or deleted) were termed as CNA-driven enhancers.

### Identification of enhancer-gene and enhancer-lncRNA pairs

Identification of differentially expressed genes: we firstly used the PAM50 algorithm (45) to classify TCGA breast cancer samples into four subtypes (Basal-like, Her2, LumA, LumB). For a given subtype, we identified subtype-specific differentially expressed genes (DEGs) and differentially expressed lncRNAs (DELs) compared to other subtypes using DESeq2, with a threshold of log fold change (logFC) and the false discovery rate (FDR) were set at 1 and 0.05, respectively. For a given gene, its expression differences across the four subtypes was calculated using one-way ANOVA with a threshold of P value < 0.05.

Enhancer-gene and enhancer-lncRNA pairs: for each breast cancer subtype, we firstly mapped genomic coordinates of subtype-specific DEGs and DELs to CNV-driven enhancers using BEDTools. Subtype-specific DEGs (or DELs) within 100 kb of CNV-driven enhancers were then selected (46, 47). Finally, upregulated genes (or lncRNAs) with an amplified enhancer and downregulated genes (or lncRNAs) with a deleted enhancer were selected as enhancer-gene (or enhancer-lncRNA) pairs.

### ELMN network construction and key module identification

We performed co-expression analysis using Spearman correlation between DEGs and DELs in each breast cancer subtype. Co-expressed lncRNA-mRNA pairs were identified using the threshold of statistical significance P < 0.05. Furthermore, we integrated CNA-driven enhancers, enhancer-genes, enhancer-lncRNAs, and lncRNA-mRNA co-expressed pairs to reconstruct subtype-specific enhancer-lncRNA-mRNA regulatory networks (ELMN) in each breast cancer subtype.

### Survival analysis

We used GEPIA2 to calculate prognostic value for lncRNAs or genes in the ELMN network (48). The expression median of the selected lncRNAs or genes were used to divide the patients with a specific breast cancer subtype into two groups. The Kaplan-Meier method and log-rank test were used to assess the difference in survival time between the two groups. Log-rank tests were used assess the differences in survival times between different groups of patients. LncRNAs or genes with P value < 0.05 were determined to be subtype-specific prognostic biomarkers.

For a lncRNA-mRNA pair, univariate cox regression analysis was used to analyze the effect of individual lncRNA and mRNA on the prognosis. A risk score was defined for each patient according to a linear combination of the expression values weighted by coefficients from univariate Cox regression analysis. The breast cancer samples were then divided into high-risk group and low-risk group according to the median value of risk scores. The prognostic value of lncRNA-mRNA pairs were determined using univariate cox regression analysis, stepwise cox regression analysis, and log-rank test analysis. LncRNA-mRNA pairs with P value<0.05 were determined to be subtype-specific prognostic biomarkers.

## Conclusions

This study characterized the effect of CNV-driven enhancers on the expression of target genes and their potential prognostic value in patients with breast cancer subtypes based on TCGA breast cancer multi-omics data. We first identified breast cancer copy number variation-driven enhancers, and then we identified breast cancer subtype specific differentially expressed genes and differentially expressed lncRNAs. In addition, by establishing the association between CNV-driven enhancers and differentially expressed genes and differentially expressed lncRNAs, we constructed the subtype-specific CNV-driven enhancer-lncRNA-gene networks for breast cancer. We identified targets of CNA-driven enhancers and enhancer-related lncRNA-mRNA pairs as prognostic biomarkers, including MUM1 and the AC012313.2-MUM1 pair in the LumA, the AC026471.4-PLK5 pair in the LumB, AC016876.1 and the AC027307.2-OAZ1 pair in the Basal-like subtype and the AC022431.1-HCN2 pair in the Her2 subtypes. Our results provided new ideas for clinical diagnosis and treatment of breast cancer patients.

## Discussion

A large number of studies have shown that CNVs not only directly affect the expression of genes but also regulate gene expression by acting on enhancers (49). In this study, we identified copy number variation-driven enhancers by integration of expression data, copy number data, and H3K27ac data and explored their effects on the expression of subtype-specific target genes in the Basal-like, Her2, Luminal A and Luminal B breast cancer subtypes. Based on subtype-specific CNV-driven enhancer-lncRNA-mRNA regulatory network mining and survival analysis, we reported that two CNV-driven enhancer target gene, four enhancer-related co-expressed lncRNA-mRNA pairs could serve as breast cancer subtype-specific prognostic biomarkers, providing new ideas for clinical diagnosis and treatment of breast cancer patients.

We constructed subtype-specific enhancer-lncRNA-mRNA networks and identified prognostic markers for each breast cancer subtype. We identified two target genes located downstream of enhancers and four lncRNA-mRNA relationship pairs as prognostic biomarkers for breast cancer subtypes. Six of these genes (MUM1, AC016876.1, PLK5, HCN2, OAZ1, AC027307.2) have been identified as prognostic features for many cancers, such as colorectal cancer, breast cancer, non-small cell lung cancer (50, 51). MUM1 and AC016876.1 were identified as independent prognostic biomarkers for LumA and Basal subtypes, respectively. In the LumA subtype, MUM1 is a risk factor, and in the Basal subtype, AC016876.1 is a protective factor. We found that MUM1 was significantly up-regulated in the LumA subtype, especially in stage II and stage III LumA patients, as compared to LumB subtype using the TCGA dataset (stage II; P=6.6e-3; stage III; P=1.5e-4; **Figures S1D, E**). To further characterize the expression of MUM1 in LumA subtype, we obtained expression data from METABRIC dataset which consists of 1904 breast cancer samples through cBioPortal. Consistent with the TCGA data, we found that the expression of MUM1 are significantly overexpressed in the LumA subtype as compared to LumB (P<0.05; **Figure S1F**). In the Basal-like subtype, AC016876.1 was significantly downregulated as compared to the other subtypes, but it was not significantly difference between Basal-like and Her2 subtypes (P=0.64; **Figure S1G**). We assessed the expression difference of AC016876.1 betwee Basal-like and Her2 patients with different clinicopathologic features. We found that AC016876.1 showed marginally significant lower expression in Basal-like than Her2 patients with uninvolved lymph node (stage ;N0; P=0.05; Wilcoxon test; **Figure S1H**). Lower expression of AC016876.1 was significantly associated with poorer prognostic outcome in the Basal-like subtype, whereas it was not found in the Her2 subtype (**Figure S1I**). In addition, we identified enhancer-related co-expressed lncRNA-mRNA relationship pairs as prognostic biomarkers for breast cancer subtypes, including one (OAZ1-AC027307.2) in Basal subtype, one (MUM1-AC012313.2) in LUMA subtype, one (PLK5-AC026471.4) in LUMB subtype, and one (HCN2-AC022431.1) in HER2 subtype. And the high risk scores of lncRNA-mRNA relationship pairs are risk factors for corresponding breast cancer subtypes. Therefore, they may be candidates for therapeutic targets in breast cancer. However, there are few large-scale multi-omics studies and clinical data on breast cancer subtypes, and our understanding of the commonality and difference of the four breast cancer subtypes is not deep enough. With the emergence of more large-scale multi-dimensional omics and clinical data of breast cancer subtypes, the ability of our method to characterize breast cancer subtypes and identify subtype-specific prognostic factors may be further improved, providing new ideas for breast cancer subtype classification and clinical treatment.

## Data availability statement

The original contributions presented in the study are included in the article/**Supplementary Material**. Further inquiries can be directed to the corresponding authors.

## Author contributions

HZ, SN and LW designed the study, implemented the algorithm, and performed the analysis. HZ, SZ, LW and LL wrote and revised the manuscript. XY, KL, LXW, LB, HX, SL, KF, WL, and MF help to collect the data and prepare the figures and tables. XY and CZ help to modify the manuscript and figures. All authors contributed to the article and approved the submitted version.

## Funding

This work was supported by University Nursing Program for Young Scholar with Creative Talents in Heilongjiang Province (UNPYSCT-2020174); Excellent Youth Project of Provincial scientific research Institute (CZKYF2022-1-C006); the Hei Long Jiang Postdoctoral Special Foundation (LBH-TZ1018).

## Acknowledgment

We are grateful to all those who contributed to this study, also thank to all the funding that provided financial support for this study.

## Conflict of interest

The authors declare that the research was conducted in the absence of any commercial or financial relationships that could be construed as a potential conflict of interest.

## Publisher’s note

All claims expressed in this article are solely those of the authors and do not necessarily represent those of their affiliated organizations, or those of the publisher, the editors and the reviewers. Any product that may be evaluated in this article, or claim that may be made by its manufacturer, is not guaranteed or endorsed by the publisher.
