# Supplementary material for: Identifying enhancer-driven subtype-specific prognostic markers in breast cancer based on multi-omics data
Source: Front Immunol. 2022 Oct 11;13:990143. doi: 10.3389/fimmu.2022.990143 (PMC9592759; doi:10.3389/fimmu.2022.990143)
Supplement: Supplementary file 2 [file DataSheet_2.docx]

**Supplementary information**

**Supplementary figures and tables**

**
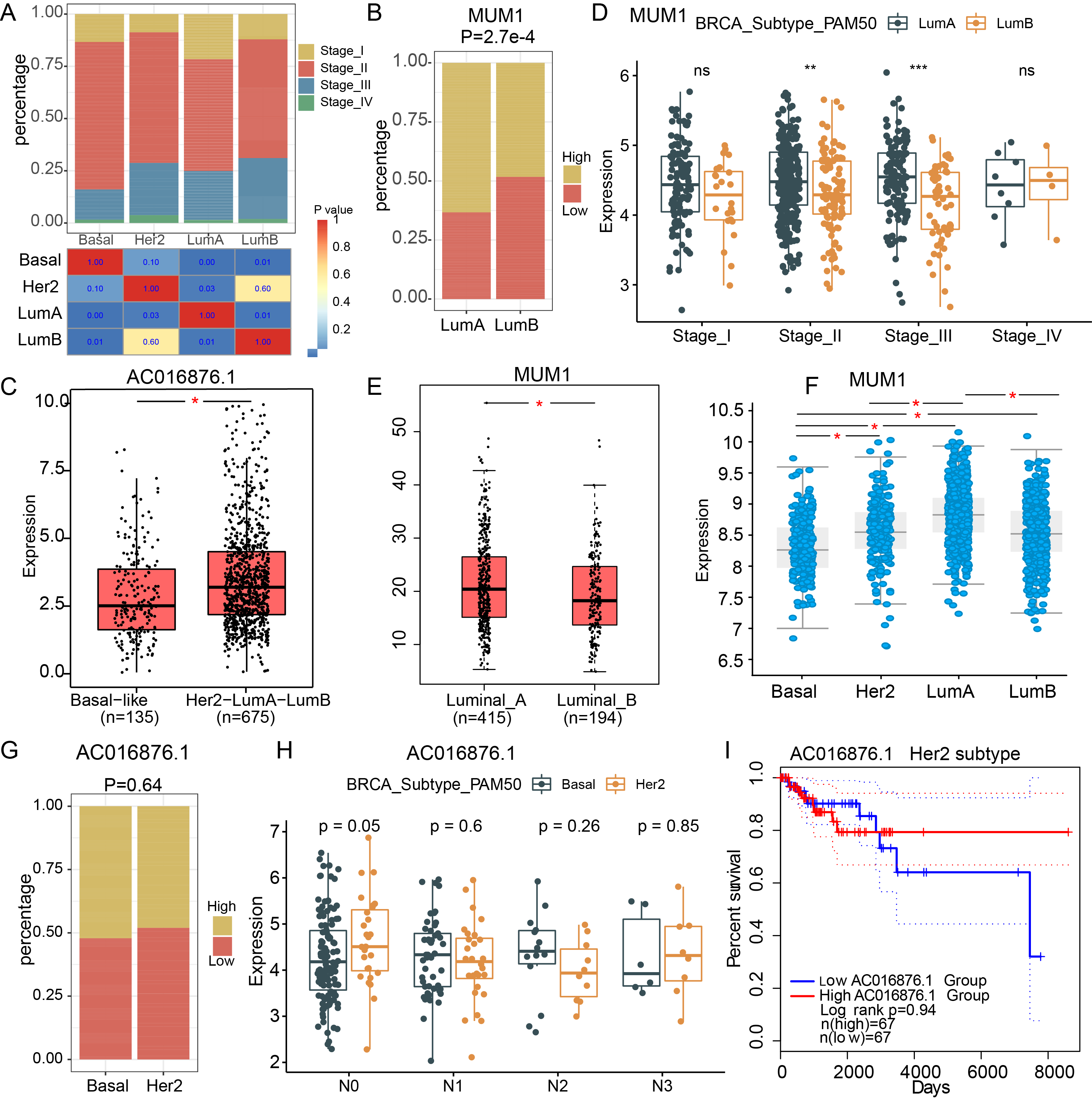
**

**Supplementary Figure S1: Correlation between prognostic markers and clinical features.** (A) Distribution of the four pathological stages of breast cancer among the four subtypes. The chi-square test was used to calculate significance. (B) Distribution of MUM1 high and low expression groups in LumA and LumB subtypes using chi-square test. (C) AC016876.1 expression levels in Basal and the other subtypes using Wilcoxon test. (D) MUM1 expression levels at different pathological stages in LumA and LumB subtypes using Wilcoxon test. (E) MUM1 expression levels in LumA and LumB subtypes using one-way ANOVA. (F) Expression levels of MUM1 among the four subtypes of breast cancer in the validation set using one-way ANOVA. (G) Distribution of AC016876.1 high and low expression groups in Basal and Her2 subtypes using chi-square test. (H) AC016876.1 expression levels at different pathological stage in Basal and Her2 subtypes using Wilcoxon test. (I) KM survival analysis of AC016876.1 in Her2 subtypes.

**Supplementary Table S1. CNA-driven enhancer-gene pairs and enhancer-lncRNA pairs in four subtypes of breast cancer.**
